# The 1287 G/A polymorphism of the Norepinephrine Transporter gene (NET) is involved in Commission Errors in Korean children with Attention Deficit Hyperactivity Disorder

**DOI:** 10.1186/1744-9081-7-12

**Published:** 2011-05-13

**Authors:** Dong-Ho Song, Kyungun Jhung, Jungeun Song, Keun-Ah Cheon

**Affiliations:** 1 Department of Child and Adolescent Psychiatry and Institute of Behavioral Science in Medicine, Severance Children's Hospital, Yonsei University College of Medicine, Seoul, South Korea; 2 Division of Child and Adolescent Psychiatry, Department of Psychiatry, Kwandong University College of Medicine, Kynggi, South Korea

## Abstract

**Background:**

Previous evidence supports the role of noradrenergic systems in ADHD, and norepinephrine transporter (NET) is critical in regulating the noradrenergic system. The present study aimed to investigate the association between NET gene polymorphism and the performance measures of the Continuous Performance Test (CPT) in Korean ADHD children.

**Methods:**

Eighty-seven children (mean age = 9.23 ± 1.99 years) with ADHD were recruited from a university hospital. Genotypes of G1287A of the NET gene (SLC6A2) were analyzed. All participants completed the CPT, with performance measures of omission errors, commission errors, reaction time and reaction standardization computed. The relationship between G1287A polymorphisms and CPT performance measures was examined.

**Results:**

There were 46 subjects with the G/G genotype, 35 subjects with the G/A genotype and 6 subjects with the A/A genotype. Among the three groups, there were no significant differences in the performance of CPTs. When dichotomized according to whether the subjects have the rare allele or not, subjects with the homozygous G/G genotype showed significantly lower commission errors compared to those without G/G genotypes (by independent T-test, t = -2.18, p = 0.026).

**Discussion:**

Our study found a significant association between commission errors of the CPT and the G1287A genotype of the NET gene in Korean ADHD children. These findings suggest a protective role of the G/G genotype of the NET polymorphisms in the deficits of response inhibition in ADHD children.

## Background

ADHD is a highly prevalent childhood psychiatric disorder that is characterized by inattention, impulsivity and hyperactivity. Neurobiological studies of ADHD point to the role of genetic factors with its high heritability estimates[1-3]. Considerable efforts have been made to investigate the molecular basis of ADHD, with most investigation on dopaminergic and noradrenergic genes[4], but results have been variable. One of the reasons that could explain the lack of consistency in the genetic studies of ADHD is the great phenotypic heterogeneity of the disorder. In an effort to overcome the limits of categorical approach to a highly heterogeneous condition, the need for investigations of endophenotypes through quantitative neuropsychological assessments have been emphasized[5-7].

Continuous performance test (CPT) is one of the most widely used neuropsychological tests in ADHD. The test assesses several measures including sustained attention in response to target stimuli and inhibitory control in response to non-target stimuli. These measures are continuously quantifiable and probabilistically predictive of ADHD[8], with recent meta-analytic review reporting that the CPT measures possess the largest effect size for the diagnosis of ADHD[9]. Due to these characteristics, the measures of CPT have recently been proposed as a promising endophenotype for ADHD[8]. Thus far, a number of studies that investigated the genetic basis of CPT have mostly focused on dopaminergic genes such as the dopamine D4 receptor gene[10,11], the dopamine D5 receptor gene[12] and the dopamine transporter gene[13]. In these studies, variants of the different dopaminergic genes have been associated with measures of inhibitory control and response time variability on CPT performance. In contrast, the investigation of the effects of the noradrenergic system has been relatively scarce.

Several lines of genetic research have suggested the role of noradrenergic pathways in the pathophysiology of ADHD. The noradrenergic system is known to be involved in the modulation of attention and arousal. Its activation has marked effects on the function of the prefrontal cortex (PFC), with particular relevance to ADHD[14]. Low to moderate concentrations of norepinephrine (NE) improves PFC function, whereas high levels of NE that can be released during stress are known to impair PFC function[14]. Clinically, the improvement of ADHD symptoms with atomoxetine, a highly selective noradrenergic reuptake inhibitor, supports the significant role of the noradrenergic system in the disorder[15,16]. Although no clear conclusion has been drawn, a number of studies have reported an association between the norepinephrine transporter gene (SLC6A2) and ADHD[17,18]. The NET SLC6A2 gene is located on chromosome 16q12.2. with a genomic structure of 14 exons spanning over 48 kb[19,20]. Among the different polymorphisms identified for the NET gene, the G1287A polymorphism at exon 9 has received attention in relation to ADHD. In a study by Yang et al.[21], ADHD subjects with the A/A genotype of the G1287A polymorphism showed less symptom reduction after treatment with methylphenidate (MPH) compared to those with other genotypes (G/A or G/G genotypes). Although several other studies that followed failed to find a significant association between G1287A polymorphism and ADHD[22,23], a recent study of Korean ADHD children showed an association between G1287A polymorphism and MPH response [24].

In regards to the CPT, only a few studies have explored the association with the NET gene. In a study by Cho et al.[23], ADHD subjects with the T allele (A/T or T/T genotypes) of the -3081 polymorphism showed a trend of higher mean score in response time variability of the CPT than those with the A/A genotype, while no differences were found between genotypes of G1287A polymorphism in the CPT performance measures. More recently, Kollins et al.[8] reported a significant association between reaction time variability of the CPT and the NET gene polymorphism (rs3785155) in 364 individuals from 152 families with at least one child diagnosed with ADHD. With mixed results, more studies are warranted to clarify the roles of the noradrenergic systems in the molecular genetic basis of ADHD. This study aimed to add to this potentially important part of the literature on the pathophysiology of ADHD by investigating the association between NET gene polymorphism and the CPT performance in children and adolescents with ADHD.

## Methods

### Subjects

Eighty-seven children with ADHD were recruited from a child psychiatric outpatient clinic in a university hospital in South Korea. Inclusion criteria were as follows: 1) subjects diagnosed as having ADHD according to the DSM-IV diagnostic criteria, 2) subjects between the ages of 6-15 years, 3) those who gave informed consent, and 4) those who have never taken psychostimulants. Exclusion criteria were as follows: 1) subjects diagnosed with autism, mental retardation, language difficulties or developmental problem including learning difficulties, 2) those with a history of a brain damage or seizure disorders, 3) those without an informed consent form from their parents or guardians. The study was approved by the Institutional Review Board. Korean version of the Kiddie-Schedule for Affective Disorder and Schizophrenia Present and Lifetime Version (K-SADS-PL) was used as a diagnostic tool. K-SADS-PL[25] is a semistructured interview for the psychiatric diagnosis of children. The Korean version of the K-SADS-PL was translated by Kim et al., and its validity and reliability have been proved to be high[26]. We measured the IQ using the WISC III (Wechsler Intelligence Scale for Children-Third Edition)[27] - Korean version.

### ADHD Rating Scale-IV (ARS)

The ARS is the ADHD symptom severity scale designed by DuPaul et al.[28] composed of a total of 18 items. Each item has a 4-point scale from 0 to 3. The 18 items are composed of 9 items reflecting the symptoms related with inattention and 9 items reflecting the symptoms related with hyperactivity and impulsivity. The Korean version of ARS was standardized.

### Continuous Performance Test

All participants completed the computerized CPT. The Korean version of the CPT used in this study was developed and standardized by Shin et al.[29]. Subjects went through a practice run before undertaking the test. Visual stimuli incorporating shapes were used, and all scores were automatically recorded in the computerized test. The test durations for children according to their age were: 1) ten minutes for those between the ages of 6-7 years, and 2) fifteen minutes for those older than seven years old. The four measures of omission errors, commission errors, the mean reaction time and the standard deviation of the reaction time (reaction time variability) were computed.

The details about four indices were as follows: 1) Number of cases where a response is missed - namely, omission error, as indicator of inattention; 2) Number of cases where a response occurs in the presence of non-target stimuli - these are commission errors, an indicator of hyperactivity or impulsivity; 3) Mean reaction time that measures the speed of process handling as a hit response time toward target stimuli; 4) Standard deviation of reaction time that measures vigilance[29]. The results were converted into values of assessments on the basis of standard computation from the normal group of the same age. The measured values were used for statistical analyses.

### Preparation of Genomic DNA and Genotyping

Genomic DNA was extracted from the blood lymphocytes of all participants with a genomic DNA extraction kit (Bioneer, Daejeon, South Korea). Detection of the SNP was done by the analysis of the primer extension products generated from the previously amplified genomic DNA using a chip-based Matrix-Assisted Laser Desorption/Ionization - Time of Flight (MALDI-TOF) mass spectrometry platform (Sequenom, Inc., CA). The general methods were performed according to the protocol of the producer.

### Polymerase Chain Reaction (PCR)

PCR primers were generated using the Primer3 program http://www-genome.wi.mit.edu/cgi-bin/primer/primer3_www.cgi. The forward and reverse PCR primers (5'- ACGTTGGATGAGACCCTAATTCCTGCACCC and 5'- ACGTTGGATGTTCAGGACCTGGAAGTCATC) were used to generate PCR products. The PCR was performed in a volume of final volume of 5 μl containing 1X PCR buffer (TAKARA, Japan), 2.5 mmol/L MgCl_2_, 0.2 mmol/L of each deoxyribonucleotide triphosphate (dNTP), 0.1 U HotStar Taq Polymerase (Quiagen GmbH, Germany), 8 pmol/L of each primers and 4.0 ng of the genomic DNA. The PCR reaction consisted of the following processes: denaturation at 95°C for 15 min, 45 cycles of 95°C for 20 sec, 56°C for 30 sec and 72°C for 1 min, with the final extension step at 72°C for 3 min.

### Homogeneous Mass EXTEND (hME)

To remove the unincorporated dNTPs, 0.3 U of Shrimp alkaline phosphatase were added and incubation was performed for 20 min at 37°C, followed by 5 minutes at 85°C to inactivate enzyme.

The oligonucleotide sequence of the hME extension primer was 5'- GCATGGAGGCTGTCATCAC, which was designed manually. We selected the forward or the reverse DNA strand depending on several factors including the characteristics of the polymorphism and the GC content. The final volume of each reaction was 9 μl, containing hME enzyme (Thermosequenase; GE Healthcare, UK), the termination mix and 5 μmol/L of the extension primer. The primer extension reaction was started with the initial denaturation at 94°C for 2 min, followed by 55 cycles of 94°C for 5 sec, 52°C for 5 sec, and 72°C for 5 sec. The reaction product was desalted with SpectroCLEAN (Sequenom, Inc., CA). The samples were distributed on 384 well SpectroCHIP (Sequenom, Inc., CA), using SpectroJET (Sequenom, Inc., CA). The SpectroCHIPs were analyzed by MALDI-TOF MassARRAY system (Bruker-Sequenom, CA). After an overall automatic measurement, assays with bad peaks were checked manually.

### Statistical Analyses

For statistical analyses, one-way analysis of variance (ANOVA) was used to evaluate the association between the three genotype groups (G/G genotypes, G/A genotypes and A/A genotypes) and the four CPT measures. Due to the small sample size of those with A/A genotypes, subjects were dichotomized according to whether they have the rare A allele or not. Independent T-test was used to compare the differences in the CPT measures between the two groups. The level of significance was held at 0.05. All analyses were done by the Statistical Package for the Social Science (SPSS; SPSS Inc., Chicago, IL, US) for Windows.

## Results

### Demographic and Clinical Characteristics

Eighty-seven ADHD children were enrolled, consisting of 72 males (82.8%) and 15 females (17.2%). The mean age of the ADHD subjects was 9.23 years of age (SD = 1.99), and the average total IQ was 104.79 (SD = 16.2). Among the 87 ADHD subjects, 39 (44.8%) were classified as ADHD combined type, 40 (46.0%) as the inattentive type and 8 (9.2%) as the hyperactive/impulsive type. The average total score of the ADHD symptom rating scale measured by the parents was 32.26 (SD = 7.91). There were no significant differences among groups of G/G, G/A and A/A genotypes in the demographic and clinical characteristics (Table 1).

**Table 1:** Demographic and clinical characteristics according to *SLC6A2 *genotype

	GG(n = 46)	GA(n = 35)	AA(n = 6)	p
Age	9.22 ± 1.89	9.14 ± 2.17	9.83 ± 1.94	.738^1)^
Sex				.167^2)^
Male (%)	40(87.0)	26(74.3)	6(100.0)	
Female (%)	6(13.0)	9(25.7)	0(0)	
IQ	106.87 ± 13.44	100.86 ± 18.31	111.83 ± 20.63	.140 ^1)^
ADHD Subtype				
Combined (% of each genotype)	18(39.1)	17(48.6)	4(66.7)	.609^2)^
Inattentive (% of each genotype)	24(52.2)	14(40.0)	2(33.3)	
Hyperactive/Impulsive (% of each genotype)	4(8.7)	4(11.4)	0(0)	
Comorbidity				
CD (% of each genotype)	0(0)	3(5.7)	0(0)	.219^2)^
ODD (% of each genotype)	1(2.2)	1(2.9)	0(0)	.908^2)^
Mood (% of each genotype)	10(21.7)	8(22.9)	0(0)	.262^2)^
Anxiety disorder (% of each genotype)	4(8.7)	5(14.3)	1(11.1)	.742^2)^
Tic (% of each genotype)	7(15.2)	0(0.0)	1(16.7)	.051^2)^
ARS scores				
Total	31.72 ± 7.97	32.89 ± 8.08	32.83 ± 7.68	.796 ^1)^
Inattentive	16.74 ± 3.96	16.83 ± 4.44	17.67 ± 3.83	.876 ^1)^
Hyperactivity/impulsivity	14.98 ± 6.81	16.06 ± 6.05	15.17 ± 5.15	.752 ^1)^

^1) ^Data determined using one-way analysis of variance test.

^2) ^Data determined by χ^2 ^test or Fisher's exact test

ADHD, Attention-deficit hyperactivity disorder; ODD, Oppositional defiant disorder; ARS, ADHD rating scale

### Genetic Polymorphisms of SLC6A2

The distribution of the genotypes for SLC6A2 was as follows: 46 subjects (52.9%) with the G/G genotype, 35 subjects (40.2%) with the G/A genotype and 6 subjects (6.9%) with the A/A genotype. The results were similar to the previous studies in Korean ADHD children (Cho et al.) The distribution of the genotypes was in Hardy-Weinberg equilibrium.

### Comparison of the CPT performance between the subjects with and without G/G genotype

**Figure 1:**
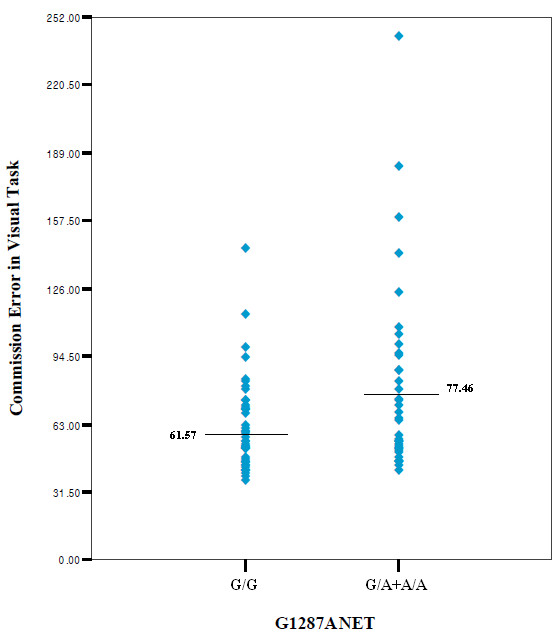


Among the three SLC6A2 genotype groups, there were no significant differences in the performance of CPTs. When dichotomized according to whether the subjects have the rare allele or not, subjects with the homozygous G/G genotype showed significantly lower commission errors compared to those without G/G genotypes (by independent T-test, t = -2.18, p = 0.026) (Figure 1). No significant differences were found in other performance measures of the CPT between the two genotypes (Table 2).

**Table 2:** Comparison of four performance measures on CPT according to G1287A genotypes of *SLC6A2*

	Mean (SD)			F	p-value^1)^	Mean (SD)		t	p-value ^2)^
						
	GG (n = 46)	GA (n = 35)	AA (n = 6)			G/G (n = 46)	G/A +A/A (n = 41)		
Omission errors	66.22 (40.44)	58.89 (18.17)	53.67 (19.71)	.755	.473	66.22 (40.44)	58.12 (18.24)	1.179	.242
Commission errors	61.57 (21.35)	78.80 (42.18)	69.66 (44.06)	2.734	.071	61.57 (21.35)	77.46 (45.02)	-2.260	.026*
Reaction time	56.04 (15.71)	52.86 (10.94)	57.50 (30.59)	.522	.595	56.04 (15.71)	53.54 (14.88)	.764	.447
Reaction time variability	67.33 (25.94)	69.80 (20.51)	75.33 (52.82)	.280	.756	67.33 (25.94)	70.61 (26.65)	-.581	.563

^1) ^By one-way ANOVA, ^2) ^By t-test, CPT: Continuous Performance Test, SD: Standard deviation, * p < 0.05

## Discussion

Our study found a significant association between commission errors of the CPT and the NET G1287A genotype in Korean ADHD children. The finding suggests that there may be a protective role of the G allele regarding the response inhibition deficit in ADHD. Furthermore, the results emphasize the possible role of the noradrenergic system underlying the pathophysiology of ADHD.

The commission errors are an indicator of deficits in response inhibition. Barkley[30] proposed response inhibition as the core deficit in ADHD, noting that it is integral to behavioral regulation and maintenance of executive function. Our data shows that the NET gene polymorphism may affect response inhibition deficits in ADHD, with G/G genotypes presenting less commission errors. This result is in consistency with the results by Yang et al. [21] in which ADHD subjects with the G allele (G/A or G/G genotypes) showed more symptom improvement compared to those with the A/A genotype in response to MPH. Together, these findings suggest a possible protective effect of the G allele in the core deficit of inhibitory control in ADHD.

Atomoxetine selectively increases catecholamine neurotransmission in the prefrontal cortex, without the associated effects of psychostimulants on striatal dopamine transmission. In the study by Chamberlain et al.[31], a single dose of atomoxetine selectively improved performance of inhibitory control, as measured by stop-signal reaction times in a stop-signal task and commission errors in the CPT, while deficits of working memory remained in adults with ADHD. These results were consistent with the animal studies in which atomoxetine reduced impulsive responses on the five-choice serial reaction time test[32] and improved stop-signal reaction times in rats[33]. Similarly, yohimbine, an alpha-2 adrenoreceptor antagonist, increased impulsivity as measured by commission errors in a task derived from CPT in healthy volunteers[34]. In neuroimaging studies, abnormalities of the right inferior frontal cortex, which is known to be associated with inhibitory dysfunction, have been shown during a test of response inhibition in medication-naïve adolescents with ADHD[35], while atomoxetine was shown to increase the activation of this region in healthy volunteers[36]. Parallel to our results at a molecular genetic level, these findings implicate the role of noradrenaline in the modulation of response inhibition.

In other lines of research, Jonsson et al.[37] reported that healthy volunteers with the G/G genotype of the G1287A polymorphism had higher cerebrospinal fluid (CSF) concentration of the NE metabolite 3-methoxy-4-hydroxyphenylglycol (MPHG) compared to other genotypes. The NET is an important component of the noradrenergic pathway which functions to reuptake NE into the presynaptic terminals. The reuptake of the dopamine in the frontal cortex is also primarily done by the NET. Considering these, abnormalities in the NET function may lead to perturbed NE and dopamine levels in the prefrontal cortex, which may in turn lead to the pathophysiology of ADHD. Furthermore, the heritability of inhibitory control measures such as the commission errors have been confirmed in both family-based and twin studies[38,39]. This indicates that the inhibitory control deficits associated with the NET gene may be heritable traits of ADHD patients and their families.

Stober et al. [40] reported that the G1287A polymorphism is a silent mutation with no functional consequences. Thus, it can be assumed that the variant probably does not have a direct effect but rather is in linkage disequilibrium with another causal variant. Kim et al. [41] reported that the G1287A polymorphism is located near SNPs, such as rs11679324, rs3285157 and rs998424, that are associated with ADHD. The authors also reported that the linkage disequilibrium is very high in these regions. Moreover, the G1287A polymorphism is located in exon 9 which encodes an uncharacterized domain of the protein placed between two transmembrane domains [40]. Thus, the affinity of the binding or the transport of neurotransmitters may be affected by this exon.

There are several limitations to be mentioned in our study. First, although the level of performance of CPT and the frequencies of the NET polymorphisms in this study are similar to previous reports, we did not compare between ADHD subjects and controls on the CPT performances and NET polymorphisms. Secondly, the sample size of the current study was very small, so the results cannot be generalized to the general population and also should be carefully interpreted. Thirdly, we did not a multiple comparison test by independent T-test to compare the four CPT measures between two groups, even though we performed a multiple comparisons test by one-way ANOVA to compare the four CPT measures among three groups. Fourthly, when we performed non-parametric test to evaluate the significance level on two group difference, even if our CPT scores were normally distributed, we found that the significance level became lower. In addition, the outlier in the G/A + A/A group might drive the more significant group difference on the commission error measure. Therefore, to eliminate some effect of the outlier of the commission error measure, we reanalyzed the comparison statistics of the commission errors between two groups without the extreme scores of the commission errors, which are considered as the outlier group in both group. However, the results still showed the significant group difference on the commission error measure. Nevertheless, we will need to perform this correlation study in the larger sample in the near future. Lastly, the differences in the male and female ratio were not controlled. Majority of participants were male in this study, and we cannot exclude the possible effects of gender differences. In future studies, these limitations should be taken into consideration to retest the current results.

In conclusion, our study found a significant association between commission errors of the CPT and the G1287A genotype of the NET gene in Korean ADHD children. The findings suggest a protective role of the G/G genotype of the NET polymorphisms in the deficits of response inhibition in ADHD children.

## Authors' contributions

KC proposed the idea of this study. KC, KJ and DS collected and evaluated the subjects with ADHD for this study. KJ and DS carried out the molecular genetic studies, and drafted the manuscript. JS and KC participated in the design of the study and performed the statistical analysis. DS participated in its design and coordination. DS and KC completed to write the final manuscript. All authors read and approved the final manuscript.

## Disclosure Statement

All authors of this work have no actual or potential conflict of interest including any financial, personal or other relationships with other people or organizations within three years of beginning the work submitted that could inappropriately influence bias our work.
